# The influence of *Jaminan Kesehatan Nasional (JKN)* on the cost of delivery services in Indonesia

**DOI:** 10.1371/journal.pone.0235176

**Published:** 2020-07-02

**Authors:** Wahyu Pudji Nugraheni, Rofingatul Mubasyiroh, Risky Kusuma Hartono

**Affiliations:** 1 National Institute of Health Research and Development, Ministry of Health, Jakarta, Indonesia; 2 Sekolah Tinggi Ilmu Kesehatan Indonesia Maju, Jakarta, Indonesia; Tulane University, UNITED STATES

## Abstract

The maternal mortality rate in Indonesia is still high, at 305 per 100,000 live births. Several studies indicated maternal financial burden as one of the dimensions of access that influence a pregnant woman’s ability to receive adequate, high-quality medical care. This study aims to identify the association between the use of Indonesia’s national health insurance (JKN) and out-of-pocket (OOP) expenditures in accessing delivery services, using data from the Indonesian Family Life Survey 5. In addition, this study also investigated the relationship of JKN and the potential reduction of catastrophic delivery expenditures (CDEs) for delivery services. The results show that JKN was associated with reduced OOP expenditures for delivery as well as reduced risk of incurring CDE. However, some OOP expenditure for cost of delivery services still exists among mothers who used JKN during delivery, potentially due to factors such as medicine stock availability and inpatient care shortages.

## Introduction

In 2015, the Indonesian maternal mortality ratio (MMR) was 305 per 100,000 live births, according to the Intercensal Population Survey (SUPAS) 2015, [1] which was far from the Sustainable Development Goals (SDGs) target of 70 deaths per 100,000 live births in 2030 [2]. Thaddeus and Maine (1994) developed the “Three Delays Models” framework that describes the causes of maternal mortality, which include: (1) the delay in deciding to seek care, (2) the delay in reaching an adequate healthcare facility, and (3) the delay in receiving adequate care once at the health facility [3]. They noted that the second delay was closely related to the financial burden mothers may face during delivery.

Many studies have used out-of-pocket (OOP) health expenditure to measure a mother’s financial burden when accessing adequate maternal healthcare [4–6]. A study in India found that high OOP payment for delivery care discouraged mothers from using facility-based delivery (FBD) care, especially for the poorest socioeconomic group [7]. In addition, evidence indicates that high OOP expenditures increase the risk of a household’s risk to experience catastrophic financial conditions [4].

Health insurance has played a critical role in reducing the patient’s financial burden when accessing care and also increases health care access. A study conducted by Bonfrer, Breebaart, & Van de Poel (2016) showed that the National Health Insurance Shceme (NHIS) in Ghana, increased access to maternal health services by 7 percent compared to before program implementation [8].

The Government of Indonesia (GOI) introduced its national health insurance program *Jaminan Kesehatan Nasional* (JKN) managed by the Social Security Organizing Agency (*Badan Pengelola Jaminan Kesehatan-BPJS*) in order to provide easy access to health services to the entire population (with a focus on targeting and subsidizing care for the poor) and improve the management of its health system. One of JKN’s objectives is to increase women’s access to high-quality maternal and neonatal health (MNH) services, with the ultimate goal of reducing Indonesia’s high MMR.

JKN aims to cover all residents, and the Presidential Regulation No. 82 of 2018 concerning health insurance requires Indonesian citizens and foreign nationals who live 6 months or more in Indonesia to register in the scheme [9]. Under this law, JKN participants are entitled to receive services in health facilities. This includes routine antenatal care and other related care. JKN implements a tiered health service system, in which sick patients are required to first visit a primary health facility (either a public health center, clinic or private practice doctors). The patient undergoes basic examination in the primary health facility and granted a referral to a hospital only if needed. Patients are only able to bypass this first primary health facility visit in an emergency. Normal delivery services are usually carried out in primary health facilities. A referral to a secondary or tertiary care facility is granted in the event of maternal complications or another amergency. The allocation by JKN to the midwives and doctors for normal delivery is paid by BPJS to primary health care facilities.

The introduction of JKN followed a major restructuring of the public health insurance system in Indonesia. JKN merged all previous public health insurance schemes, including their benefits and coverage of the MNH continuum of care (e.g., antenatal care, delivery, and postnatal care). JKN now provides a non-capitation budget limit for all type of delivery services which depend on facility used during delivery. JKN allocates IDR. 600,000 for normal delivery assisted by midwife; a maximum of IDR 700,000 for deliveries assisted by doctor; and a maximum IDR 950,000 for emergency delivery services at a primary health care facility.

In addition, JKN applies the Indonesian Case-Based Groups (INA-CBGs) which is a claim payment by BPJS Health to advanced referral health facilities (hospitals that work with BPJS Health) based on grouping disease diagnoses and procedures [10]. Implementation of INA-CBG rates varies by severity, regional hospital areas, hospital classes, and hospital ownership in diagnosing the same. For example an outpatient diagnosis in Region 1 (Banten, DKI Jakarta, West Java, Central Java, DI Yogyakarta, East Java Province) with vaginal delivery procedures/birth canal with CBGs O-7-13-0 in private type A hospitals is IDR 1,358,200 and IDR 896,700 at Government hospitals type B [10].

Despite the introduction of national health insurance, private insurance still plays an important role in providing financial protection for households in Indonesia. The role of private insurance is regulated (BPJS Regulation No: 4/2016) as a supplement to JKN though the coordination of benefit (COB) mechanism, which allows patients to use private insurance for any additional costs not covered by JKN, including any costs that exceed JKN’s budget limit [11]. For example, patients may need to use private insurance to pay for medicines that are not available in the National Formulary list (Fornas) or to pay for upgrades to their hospital class.

Several studies have explored the role of JKN in reducing OOP health expenditure in Indonesia [12,13]. One study found that many JKN-insured patients reported high charges at the point of service, mostly for medicine [14]. Another study found that insurance for civil servants and the poor (which later were merged into JKN in 2014), significantly reduced OOP health payments [15]. One last study from 2012 found that health insurance ownership reduced household OOP health payments by 12.97 percent [16].

Although the literature has not investigated the incidence of catastrophic delivery expenditure (CDE) for MNH services in Indonesia, such studies have been common in India [7,17–19]. These studies examine catastrophic payment for MNH services, well as correlations of these payments. One study found that the risk of CDE in India decreased with economic status but increased with education [20]. The risk of CDE was also found to increase in urban areas compared to rural areas and in private facilities compared to public facilities [21]. Another study found that health insurance was a strong determinant for the size of OOP health expenditures on MNH services [18].

This study aims to address the gap in the literature on OOP expenditure for delivery services and the risk of CDE in Indonesia. Specifically, we aim to understand whether participation in JKN reduces CDE or OOP expenditure for delivery services.

## Methods

This study used data from the Indonesian Family Life Survey 5 (IFLS-5) focusing on the Child Health module. The IFLS is an ongoing longitudinal survey that began its data collection in 1993, consisting of household, individual, and community data, representing 83 percent of the country’s total population (from a total of 13 of Indonesia’s 27 provinces). The total IFLS-5 sample includes 16,204 households and 50,148 individuals with data collected from October 2014 to August 2015 [22].

The IFLS-5 includes a total of 5,404 mothers ages 15–49 who had given birth to their last child one year before the survey period. The one-year cut-off strategy was used to reduce mothers’ potential recall bias in answering questions related to use of MNH services, OOP for delivery expenditures, insurance used for delivery, and household expenditures. This strategy, as shown by Bonu et al. (2009), allows us to reduce the timeline gap for each question and increase the quality of our results [7]. After the inclusion process, we obtained a final study sample of 2,143 mothers.

### Measures

#### Outcome variables

This study used three total outcome variables: the first was OOP expenditure for delivery services (numeric, in Indonesian Rupiahs [IDR]); the other two were binary outcome variables for CDEs, as measured by OOP expenditure for delivery services in relation to total annual household expenditure, with varing thresholds. The information on OOP expenditure for delivery services was available directly from the IFLS-5 pregnancy history section. The IFLS-5 pregnancy history section contained data on OOP expenditure for delivery services, which was defined as all costs that the mother paid for childbirth, including the costs of services, medication, and hospitalization. We also needed additional information on household expenditure which was not provided in this section. Thus, we merged the mother’s pregnancy history with her corresponding household information and retrieved household expenditures to calculate our CDE variables using household identifier provided in IFLS-5.

As seen in other living standards measurement surveys, household expenditures were reported in the IFLS-5 through three recall periods, depending on the good or service period: weekly, monthly, or annually. Short recall periods of one week were used for daily consumption expenses, such as food and drink, whereas longer recall periods were used for large and rare expenses, such as housing and the cost of purchasing needed assets. To calculate household expenditures overall as well as household non-food expenditures, we annualized weekly and monthly expenditures. Household non-food expenditures simply excluded expenditures on food.

The inclusion of two different CDE binary outcome variables served to facilitate a sensitivity analysis on different measures of financial burden. The operational definition of each CDE variable was as follows:

- **CDE 1** was a binary outcome variable, coded as 1 if OOP expenditure for delivery services were greater than (≥5) percent of total annual household expenditure (total annual household expenditure did not include OOP expenditure for delivery services) and as 0 if otherwise.
- **CDE 2** was a binary outcome variable, coded as 1 if OOP expenditure for delivery services were greater than (≥10) percent of total annual household expenditure (total annual household expenditure did not include OOP expenditure for delivery services), and 0 if otherwise.

#### Explanatory variable and covariates

The explanatory variable of interest was the type of insurance the mother used at delivery, as reported in the pregnancy history section of the IFLS-5. The type of insurance was categorized as: (1) without insurance, (2) JKN *(Asuransi Kesehatan*, *Jaminan Sosial Tenaga Kerja*, *Jaminan Kesehatan Masyarakat*, *Jaminan Kesehatan Daerah*, *Jaminan Kesehatan Nasional*, *Jaminan Persalinan Nasional)*, and (3) non-JKN (all insurances other insurance excluding JKN). Since the introduction of JKN merged members from the government employee health insurance, Jamkesmas insurance, private worker health care insurance (JPK-Jamsostek), national army and police health insurance, and local government insurance (Jamkesda), we included those categories in the JKN definition above.

This analysis also included individual, household, and geographic characteristics from the IFLS-5 as covariates, which could explain differences in the financial burden of OOP for cost of delivery expenditures.

Individual characteristic covariates included: (1) mother’s age at delivery, (2) mother’s education, (3) place of delivery, (4) mother’s occupation, and (5) presence of pregnancy complications (swelling of the feet or leg; difficulty of vision during day; difficulty of vision during night; vaginal bleeding; fever; convulsion and fainting; labor before 9 months.)

Household characteristic covariates were as follows: (1) household head’s education, (2) household head’s activity, (3) number of household members, and (4) household socioeconomic quintile.

Geographic covariates were as follows: (1) residential sites and (2) region.

### Statistical analysis

**Fig 1:**
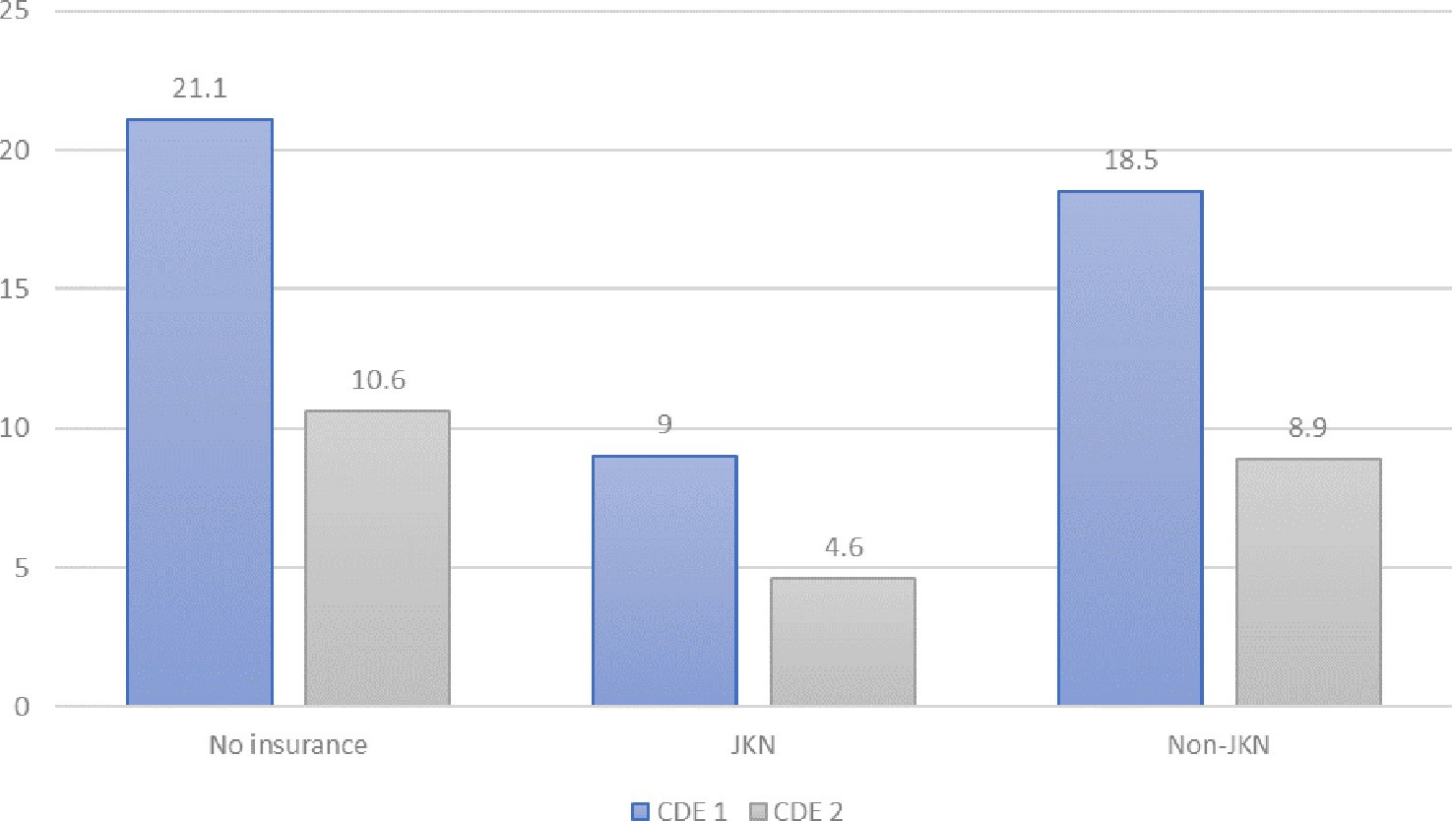
Incidence of CDE 1 and CDE 2 by mother’s insurance type.

We used descriptive statistics to illustrate outcomes and sample characteristics. The characteristics of the sample were described by frequency distribution (Table 1). Table 2 illustrated the mean of OOP expenditure for delivery services by mother’s characteristics. The catastrophic conditions of labor experienced by the mother were presented in Figs 1 and 3. We used multivariate regression analysis to explore the relationship between the outcomes and the explanatory variable. We used linear regression to explore the relationship between OOP expenditure for delivery services and type of insurance (Table 3). We normalized the OOP expenditure for delivery services by applying the natural log in the multivariate analysis. We used logistic regression for the two CDE outcome variables to identify their relationship with the insurance used for delivery. We conducted all analyses using STATA version 14.0.

**Table 1:** Characteristics of sample, by factors and covariates.

Variables	N (2,143)	%
**Type of health insurance used for delivery services**		
No insurance	1,263	58.9
JKN	756	35.3
Non-JKN	124	5.8
**Place of birth delivery services**		
Home	361	16.8
Polindes/midwife private	806	37.6
Public health centre	191	8.9
Private health centre	102	4.8
Public hospital	310	14.5
Private hospital	373	17.4
**Mother’s education**		
Primary or less	432	20.2
Junior high school	528	24.6
Senior high school	820	38.3
Diploma or more	363	16.9
**Mother’s employment status**		
Does not work	1,564	73.0
Working	579	27.0
**Mother’s age at last delivery**		
15–24 years old	672	31.4
25–34 years old	1,140	53.2
35+ years old	331	15.4
**Presence of pregnancy complications**		
No	1,671	78.0
Yes	472	22.0
**Household head education**		
Primary or less	726	33.9
Junior high school	427	19.9
Senior high school	710	33.1
Diploma or more	280	13.1
**Household head’s employment status**		
Does not work	324	15.1
Working	1,819	84.9
**Number of household members**		
More than 4	815	38.0
4 or less	1,328	62.0
**Household wealth ranking**^**a**^		
Quintile 1: Poorest	277	12.9
Quintile 2	405	18.9
Quintile 3	471	22.0
Quintile 4	504	23.5
Quintile 5: Richest	486	22.7
**Residential sites**		
Rural	897	41.9
Urban	1,246	58.1
**Region**		
Sumatra	590	27.5
Java	1,016	47.4
Bali & Nusa Tenggara Timur (NTT)	317	14.8
Kalimantan	111	5.2
Sulawesi & Papua	109	5.1

^a^Household expenditure quintiles

**Table 2:** Mean OOP expenditure for delivery services.

Variables	Mean (IDR)
**Type of health insurance used for delivery services**	
No insurance	1,946,595
JKN	887,603
Non-JKN	2,315,137
**Place of delivery services**	
Home	572,042
Polindes/midwife private	723,408
Public health centre	204,461
Private health centre	3,080,000
Public hospital	1,861,560
Private hospital	4,549,020
**Quintile**	
Poorest	594,435
Poor	972,091
Middle	1,282,355
Rich	1,697,671
Richest	2,877,950
**CDE 1**	
No	705,114
Yes	6,042,913
**CDE 2**	
No	1,000,877
Yes	8,066,294
**Average**	1,594,332

**Table 3:** Multivariate investigation of the effect of health insurance used and covariates during delivery on OOP expenditure for delivery services (IDR).

Variables	(1)	(2)
	ln OOP delivery services (IDR)	
	coefficient	SE
**Type of health insurance used on delivery services**		
No insurance (ref)		
JKN	-4.49***	(0.25)
Non-JKN	-4.91***	(0.60)
**Place of birth delivery services**		
Home (ref)		
Polindes/midwife private	1.22***	(0.25)
Public health centre	-0.73	(0.45)
Private health centre	2.33***	(0.40)
Public hospital	1.36***	(0.40)
Private hospital	2.50***	(0.34)
**Mother’s education**		
Primary or less (ref)		
Junior high school	-0.15	(0.27)
Senior high school	-0.42	(0.28)
Diploma or more	0.31	(0.39)
**Mother’s employment status**		
Does not work (ref)		
Working	-0.12	(0.22)
**Mother’s age at delivery**		
15–24 years old (ref)		
25–34 years old	-0.12	(0.21)
35+ years old	0.13	(0.31)
**Presence of pregnancy complications**		
No (ref)		
Yes	-0.02	(0.23)
**Household head’s education**		
Primary or less (ref)		
Junior high school	-0.46*	(0.27)
Senior high school	-0.26	(0.25)
Diploma or more	-0.77*	(0.41)
**Household head’s employment status**		
Does not work (ref)		
Working	-0.03	(0.27)
**Number of household members**		
More than 4 (ref)		
4 or less	-0.42**	(0.21)
**Household wealth ranking**		
Quintile 1: Poorest (ref)		
Quintile 2	0.45	(0.33)
Quintile 3	0.34	(0.35)
Quintile 4	0.86**	(0.34)
Quintile 5: Richest	0.80**	(0.37)
**Residential sites**		
Rural (ref)		
Urban	-0.15	(0.20)
**Region**		
Sumatra (ref)		
Java	-0.34	(0.24)
Bali & NTT	-0.24	(0.32)
Kalimantan	0.57	(0.38)
Sulawesi & Papua	-2.20***	(0.56)
Constant	12.82***	(0.44)
Observations	2,143	
R-squared	0.25	

Robust standard errors in parentheses

*** p<0.01

** p<0.05

* p<0.1

## Results

Table 1 illustrated the characteristics of the sample in this study. Most mothers in the sample lived in urban areas (58%), had an education level of high school or more (55%), gave birth between the ages of 25 and 34 (53%), were not working (73%), lived in a small family (62%), and lived in the Java and Sumatra regions (75%). Table 1 also shows that the use of insurance for delivery was low, with more than 50 percent of mothers in the sample not using insurance for delivery.

In addition, the use of JKN insurance at delivery was six times higher than for *non-JKN* insurance (less than 6 percent). The used of FBD by mothers in the sample was quite high, with less than 17 percent of mothers delivering at home. The proportion of mothers who delivered at a Polindes (midwife private clinic) was the largest, at 38 percent. It was followed by FBD in hospitals and health centres with, less than 20 percent for both.

### OOP expenditure for delivery services

Table 2 shows the average amount of OOP expenditures paid for delivery services. On average, the OOP expenditure were IDR 1,594,332 and varied by the mothers’ characteristics and type of insurance used. On average, the largest OOP expenditures were incurred by mothers who used non-JKN insurance (IDR 2,315,137), which was slightly higher than the payments made by mothers who did not have insurance (IDR 1,946,595). Mothers who utilized JKN reported the lowest OOP payments (IDR 887,603)―less than half of the OOP payments made by mothers without insurance.

Table 2 also shows that the amount of OOP expenditure for delivery services seems *positively associated* with mother’s choice of place of delivery. Overall, delivery in private health institutions (both in health centres and hospitals) was associated with mothers having higher OOP expenditure for delivery services, at IDR 3,080,000 and IDR 4,549,020, respectively. The lowest OOP expenditures were paid by mothers who delivered at a public health centre, with an average cost of IDR 204,461. CDE 1 was experienced by 16.7% of mothers, while CDE 2 was experienced by 8.4% of mothers. Table 2 also shows that mean OOP payments for delivery by mothers who experienced CDEs were higher than the mean of OOP expenditures in all quintiles. For example, the average OOP expenditure for delivery services for mothers who experienced CDE 1 was IDR 6,042,913; which was double the average OOP payments for mothers in the richest quintile.

Table 3 displays the multivariate linear regression results, highlighting that mothers who used any type of insurance experienced the reduction of the amount of delivery service costs significantly compared to the mothers who did not use insurance. The biggest reduction was in the use of non-JKN insurance category, which reduced the amount of OOP expenditure for delivery services by 99.2 percent (The coefficient of the log-linear result could be interpreted as a percentage change using the following formula: $\%\Delta OOP=100(e^{\beta_{1}}-1)$. See [23], pp. 142–144. The interpretation of all of the results presented in Table 4 uses the same formula) followed by use of JKN insurance (98.7%).

**Table 4:** Multivariate investigation of the effect of health insurance used and covariates for delivery on OOP expenditure for delivery services (IDR).

Variables	(1)	(2)	(3)	(4)
	CDE 1: 5%		CDE 2: 10%	
	AOR	CI (95%)	AOR	CI (95%)
**Type of health insurance used for delivery services**				
No insurance (ref)	1.00		1.00	
JKN	0.09***	(0.06–0.14)	0.11***	(0.06–0.18)
Non-JKN	0.24***	(0.13–0.45)	0.29***	(0.14–0.61)
**Place of birth delivery services**				
Home (ref)	1.00		1.00	
Polindes/midwife private	2.13**	(1.19–3.82)	2.13	(0.54–8.47)
Public health centre	0.29	(0.04–2.12)	2.00	(0.21–18.99)
Private health centre	28.52***	(14.21–57.23)	57.41***	(15.28–215.77)
Public hospital	48.75***	(24.81–95.79)	135.62***	(38.11–482.55)
Private hospital	81.71***	(42.40–157.46)	192.23***	(54.00–684.28)
**Mother’s education**				
Primary or less (ref)	1.00		1.00	
Junior high school	0.92	(0.57–1.48)	0.77	(0.41–1.45)
Senior high school	0.93	(0.59–1.46)	0.75	(0.41–1.35)
Diploma or more	1.14	(0.64–2.05)	0.65	(0.31–1.40)
**Mother’s emplyoment status**				
Does not work (ref)	1.00		1.00	
Working	0.74*	(0.53–1.05)	0.54**	(0.34–0.87)
**Mother’s age at delivery**				
15–24 years old (ref)	1.00		1.00	
25–34 years old	1.34*	(0.97–1.85)	1.16	(0.75–1.79)
35+ years old	1.05	(0.66–1.66)	0.75	(0.41–1.39)
**Presence of pregnancy complication**				
No (ref)	1.00		1.00	
Yes	1.05	(0.73–1.51)	0.84	(0.52–1.33)
**Household head’s education**				
Primary or less (ref)	1.00		1.00	
Junior high school	1.18	(0.75–1.85)	1.20	(0.67–2.17)
Senior high school	1.33	(0.87–2.02)	0.93	(0.53–1.62)
Diploma or more	1.89**	(1.10–3.25)	2.17**	(1.07–4.40)
**Household head’s employment status**				
Does not work (ref)	1.00		1.00	
Working	1.12	(0.73–1.71)	1.47	(0.82–2.62)
**Number of household members**				
More than 4 (ref)	1.00		1.00	
4 or less	0.72**	(0.53–0.99)	1.02	(0.67–1.55)
**Household wealth ranking**				
Quintile 1: Poorest (ref)	1.00		1.00	
Quintile 2	0.41***	(0.25–0.68)	0.38***	(0.18–0.77)
Quintile 3	0.21***	(0.13–0.36)	0.22***	(0.11–0.44)
Quintile 4	0.14***	(0.08–0.25)	0.25***	(0.12–0.51)
Quintile 5: Richest	0.08***	(0.04–0.15)	0.07***	(0.03–0.15)
**Residential sites**				
Rural (ref)	1.00		1.00	
Urban	0.95	(0.68–1.32)	1.01	(0.64–1.58)
**Region**				
Sumatra (ref)	1.00		1.00	
Java	1.01	(0.71–1.45)	1.64**	(1.02–2.64)
Bali & NTT	0.37***	(0.21–0.66)	0.49*	(0.22–1.08)
Kalimantan	1.50	(0.80–2.82)	2.29*	(0.89–5.87)
Sulawesi & Papua	0.39**	(0.17–0.90)	0.44	(0.14–1.40)
Constant	0.16***	(0.07–0.34)	0.02***	(0.00–0.07)
Observations	2,143		2,143	

Robust CI in parentheses

*** p<0.01

** p<0.05

* p<0.1

We observed similar associations between our factors and covariate variables with OOP expenditure as in the previous descriptive analyses. Apart from insurance used, the place of delivery also played an important role on how much money mothers spent for delivery. Mothers who delivered their baby at health facilities incurred higher OOP expenditures on delivery compared to women who delivered at home. Overall, delivery at a private health facility required mothers to spend more money. For example, after controlling for all covariates, delivery at a public hospital required a 289 percent higher OOP delivery payment (compared to delivery at home). However, this percentage was five times lower than that of mothers who delivered their baby at a private hospital.

### Catastrophic delivery services expenditure (CDEs)

Fig 1 shows the relationship between mothers who experienced CDE and their use of insurance for delivery. Overall, CDEs were more likely to be experienced by mothers who did not use insurance during delivery (21.1%). Meanwhile, a total of 18.5 percent of mothers who experienced CDE 1 used non-JKN insurance for delivery. JKN, which functions as part of the GOI’s poverty reduction program, seemed to perform better than non-JKN insurance: only 9 percent of mothers who used JKN experienced CDE the lowest compared to other types of insurance.

**Fig 2:**
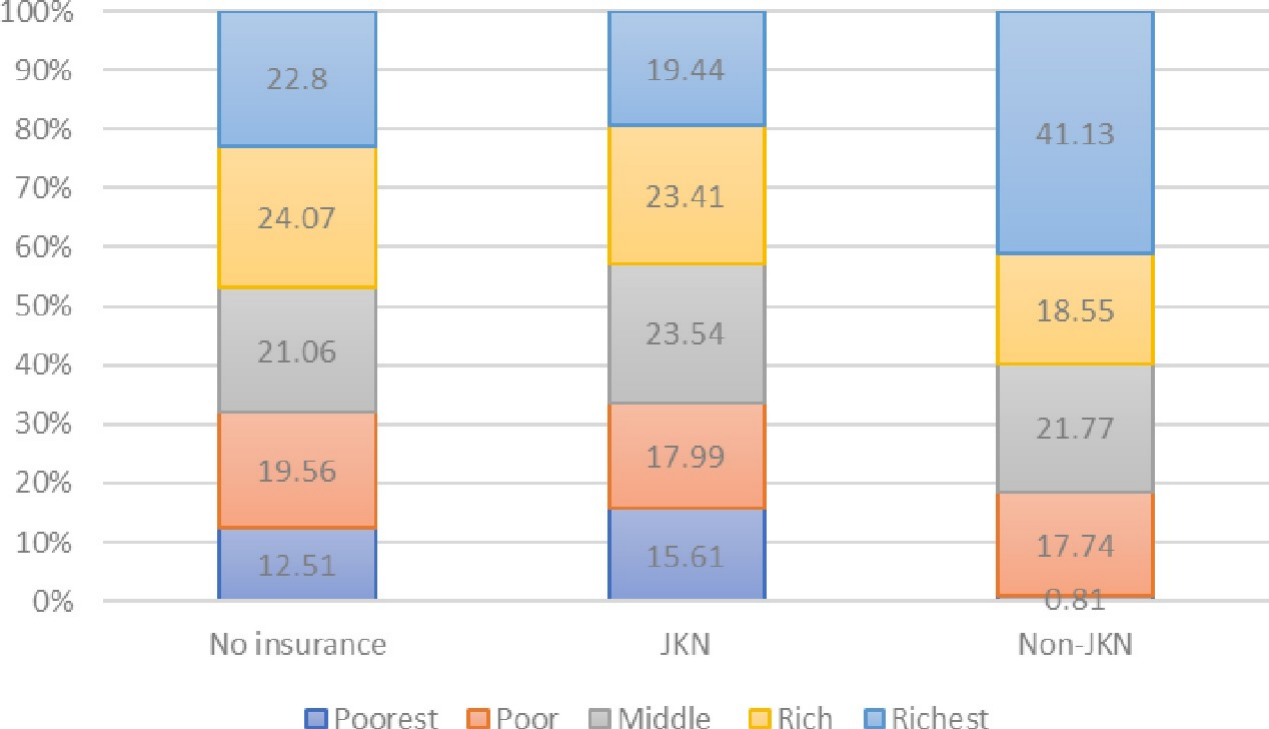
Use of insurance, by wealth status.

We further analyzed the distribution of insurance used by wealth status (Fig 2). Overall, the proportion of insurance used varied by wealth status. Non-JKN insurance was used mostly by the richest quintile, at 41.1 percent. On the other hand, JKN insurance was used mostly by the middle and rich quintiles, at 46.9 percent. The poor and poorest quintiles, which are the target beneficiaries of JKN program, used JKN the least—only 33.6 percent of the poor and poorest utilized JKN for delivery.

**Fig 3:**
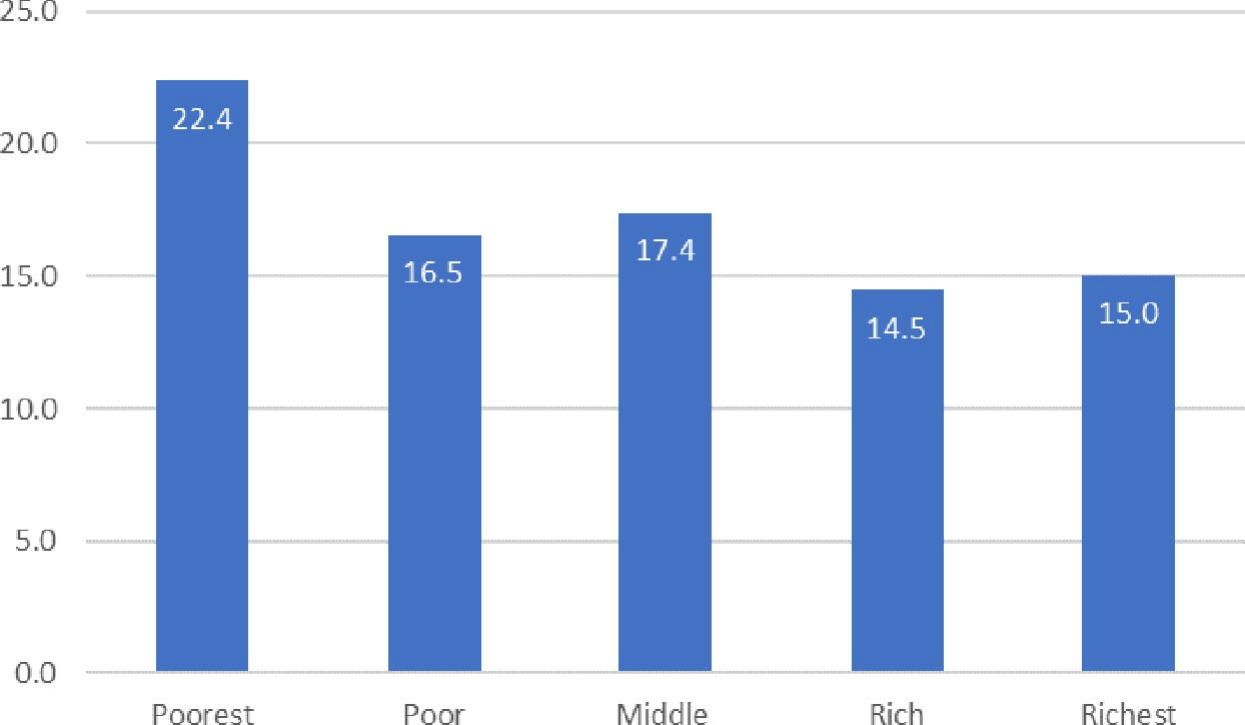
The proportion of mothers who experienced CDE 1, by wealth status.

Fig 3 illustrates the proportion of mothers who experienced CDE 1 by quintile, highlighting a negative association between CDE 1 and wealth status. As wealth status increased, the proportion of mothers who experienced CDE 1 decreased. The proportion of mothers who experienced CDE 1 in the poorest wealth quintile was 22.4 percent, whereas this proportion decreased to 15 percent among the richest mothers. These findings indicate that JKN was associated with a low prevalence of CDE 1 but had not been used fully by the poor and poorest, who bear the greatest burden from OOP expenditure for delivery services compared to any other quintile.

Table 4 presents the results of a multivariate logistic regression for different CDEs, which support the results of the linear regression shown in Table 3. Each insurance type, JKN and non-JKN, significantly influenced prevalence of CDE 1 and CDE 2. Use of JKN was associated with the lowest odds (0.09), which means that mothers who used JKN experienced a lower risk of CDE 1 compared to mothers who did not use insurance. Although offering a less protective effect, use of non-JKN insurance was still associated with the reduction in risk of CDE 1 by 76 percent (OR 0.24).

Table 4 also highlights the importance of the place of delivery in influencing the risk of catastrophic delivery services expenditure. Delivery at a public health centre was associated with a significantly lower probability of experiencing all types of CDEs compared to mothers delivering at home. For example, mothers who delivered at a private hospital experienced an 81.7 times higher probability of experiencing CDE 1 compared to mothers who delivered at home. This number was 1.6 times higher than for mothers with the same characteristics who decided to deliver at a public hospital. In addition, table 4 shows that mothers from the poorest households bore the highest economic burden of OOP expenditure for delivery services, as they experienced the highest probability of incurring CDE 1 compared to other groups.

## Discussion

Our findings may be summarized as follows: *first*, our results indicate that mothers still needed to pay OOP for delivery even though they use JKN insurance; *second*, the used of JKN for delivery was significantly associated with the reduction of both the amount of OOP expenditures for delivery services and the risk of CDE; and *third*, JKN may still face implementation challenges because the poorest group does not utilize it optimally.

The first finding highlights that having JKN did not eliminate OOP expenditure for delivery services in Indonesia completely. This finding in line with several studies in other countries, in which OOP expenditure was still observed for use of MNH services, even among those who used health insurance [5,6]. One possible explanation for our findings is that there may be shortages in supply for certain components covered by JKN in the health facilities, including drugs purchased outside the hospital (8.3%), drugs purchased inside the hospital (4.8%) and administrative costs (1.3%) [24]. For example, a patient might need to buy a required medicine outside of the health facility, which adds an additional OOP cost because it is not reimbursed or covered under JKN. Another possibility for OOP costs despite use of JKN may be due to limited availability of rooms or beds covered under JKN at the time of a mother’s delivery due to high demand [25]. This situation may force mothers either to go to another health facility or take a higher-level room or bed which might not be covered by JKN, leading to OOP costs.

We also found that mothers who used JKN insurance experinced reduced OOP expenditure for delivery services and reduced risk of CDE. One possible explanation is that JKN covers all items needed for deliveries (but still under JKN budget limit); thus JKN reduces most OOP payments and lowers the risk of CDE [26].

The government of Indonesia (GOI) designed JKN to cover multiple health expenditure including the costs for doctors, medicine, rooms, and so on. Aji et al. (2017) showed that the use of JKN could reduce OOP expenditure and CDE [15].

Last, we found the implementation of JKN is still weak and could be improved, especially for the poor. The GOI’s objective in designing JKN was to protect the poor from health risks by subsidizing insurance to them. However, our findings indicate that the poorest group does not utilize JKN for delivery services optimally, as only 15 percent of mother in the poorest group used JKN to cover their delivery services expenditure. We found OOP expenditure for delivery services in our study beyond mothers’ ability to pay might increase their risk of falling into poverty [7]. We observed that the poorest group had a higher risk of CDE but also used JKN the least of all quintiles. The average OOP expenditure for delivery services paid by the poorest quintile was IDR 594,435, whereas that paid by mothers who experienced CDE 1 was IDR 6,042,913. This finding illustrates that the poorest mothers have a higher risk of incurring OOP expenditure and a higher burden of experiencing CDE than the risk faced by those in other quintiles. One possible explanation for the poor’s low utilization of JKN, despite carrying a higher burden of expenditures is that the complicated procedures involved in JKN might hinder the poorest from optimally using JKN. For example, JKN requires a referral from a primary healthcare facility in order to cover treatment in a hospital. This referral process may hinder the poorest from using JKN covered healthcare [27]. Furthermore, because mothers still paid some OOP expenditure for delivery service, it may discourage them from using institutional care delivery and they may instead choose to deliver at home―especially relevant for those in the poorest quintile [7].

This study had several limitations. *First*, the type of insurance used and its relationship to our variables outcomes might cause us to underestimate the result because the IFLS-5 captured only the early stages of JKN implementation. Thus, our study may underestimate the actual influence of the insurance on OOP and CDE because the program was not mature enough at the time of IFLS-5 data collection. *Second*, our first limitation also presents an additional issue regarding our “operational” definition of JKN and non-JKN insurance. JKN was implemented in 2014, in the middle of the IFLS-5 survey period. In addition, the IFLS-5 collected information on the type of insurance used both before and after JKN was implemented. To mimic how JKN would have been used in the period before it was implemented, we grouped together those insurance plans that eventually were amalgamated into JKN *(Asuransi Kesehatan*, *Jaminan Sosial Tenaga Kerja*, *Jaminan Kesehatan Masyarakat*, *Jaminan Kesehatan Daerah*, *Jaminan Kesehatan Nasional*, *Jaminan Persalinan Nasional)*. This strategy was not perfect but was the best method we found. *Third*, IFLS-5 did not collect information specially on why mothers paid OOP for delivery services. This information is important for capturing the real reason why OOP expenditure for delivery services still exist and might capture mothers’ preferences for using insurance.

## Conclusion

This study revealed that the amount of OOP for childbirth is much lower for mothers who use JKN compared to other groups. In addition, this study finds that JKN was effective in reducing the amount of OOP expenditure for delivery services and the risk of CDE. Despite JKN’s design to ensure all possible delivery costs are covered, our study finds that mothers who used JKN for delivery still incurred OOP expenditures. In addition, we found that it is likely that challenges persist in the implementation of JKN, especially with its targeting of the poor, since the use of JKN by the poorest group was the lowest compared to other groups. This low usage might increase the poor’s risk of falling further into poverty because they bear the heaviest burden of all quintiles for delivery costs.

Based on results of this study, we offer several policy recommendations. *First*, the availability of and access to health insurance should cover all mothers, regardless of their socioeconomic status. As the study shows, JKN is a good tool for reducing delivery expenditures, but its effectiveness needs to improve. We recommend an evaluation of why the poor do not use JKN for delivery services. Another question that needs to be answered is how the poor pay for delivery services if they are not using JKN. This information would provide a greater understanding of the problem and help in the design of a better JKN policy that could protect the poor from the impact of delivery services expenditures. *Second*, the readiness of health facilities and practitioners to deliver adequate services based on the JKN standard may need to be improved. We also need further research on why there are some supply shortages at several health institutitons. It is possible that supply shortages are related to difficulties faced by facilities in claiming the expenses they incur. Research to answer questions raised by this study is a necessary step in improving JKN in the future.
